# Structure and Inhibition of Mouse Leukotriene C_4_ Synthase

**DOI:** 10.1371/journal.pone.0096763

**Published:** 2014-05-08

**Authors:** Damian Niegowski, Thea Kleinschmidt, Shabbir Ahmad, Abdul Aziz Qureshi, Michaela Mårback, Agnes Rinaldo-Matthis, Jesper Z. Haeggström

**Affiliations:** Division of Chemistry 2, Department of Medical Biochemistry and Biophysics, Karolinska Institutet, Stockholm, Sweden; Università degli Studi di Milano, Italy

## Abstract

Leukotriene (LT) C_4_ synthase (LTC4S) is an integral membrane protein that catalyzes the conjugation reaction between the fatty acid LTA_4_ and GSH to form the pro-inflammatory LTC_4_, an important mediator of asthma. Mouse models of inflammatory disorders such as asthma are key to improve our understanding of pathogenesis and potential therapeutic targets. Here, we solved the crystal structure of mouse LTC4S in complex with GSH and a product analog, S-hexyl-GSH. Furthermore, we synthesized a nM inhibitor and compared its efficiency and binding mode against the purified mouse and human isoenzymes, along with the enzymes’ steady-state kinetics. Although structural differences near the active site and along the C-terminal α-helix V suggest that the mouse and human LTC4S may function differently *in vivo,* our data indicate that mouse LTC4S will be a useful tool in future pharmacological research and drug development.

## Introduction

**Figure 1:**
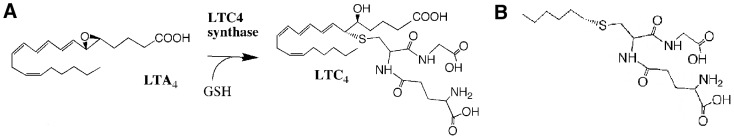
The LTC_4_ synthase reaction. A. Schematic drawing of the catalytic reaction of LTC4S where the allylic epoxide LTA_4_ is conjugated with GSH at C6, to form LTC_4_. B. Structure of the product analog S-hexyl GSH.

LTC4S is a trimeric integral membrane protein with a monomeric size of 18 kDa. It is located in the outer leaflet of the nuclear envelope and in the endoplasmic reticulum. The enzyme catalyzes the formation of the pro-inflammatory lipid mediator LTC_4_ ((5S)-hydroxy-(6R)-S-glutathionyl-7,9-*trans*-11,14-*cis*-eicosatetraenoic acid) (Figure 1A), the parent compound of the cysteinyl-leukotrienes C_4_, D_4_, and E_4_ that are established mediators of human asthma [1], [2]. The formation of LTA_4_, from membrane-derived arachidonic acid, is catalyzed by 5-lipoxygenase and Five Lipoxygenase Activating Protein (FLAP). LTA_4_ can then further be converted to LTB_4_ by LTA_4_ hydrolase, or conjugated with GSH by LTC4S to form LTC_4_ [1], [3].

The expression of LTC4S, as well as LTC_4_ production, is limited to haematopoietic cell types whereas the G-protein coupled receptors, CysLT1 and CysLT2, that mediate the signaling are more widespread. Today, CysLT1 receptor antagonists are used in the clinical management of asthma [4], [5], [6]. Unfortunately, not all patients respond to this treatment [7] and there is a need for more efficient therapeutic agents. Recent data indicate that at least five different G-protein coupled receptors can transmit cysteinyl-leukotriene signaling, although with various affinity. Therefore the biosynthetic enzyme LTC4S has emerged as a promising drug target [8].

LTC4S belongs to the superfamily of membrane-associated proteins in eicosanoid and glutathione metabolism (MAPEG), which consists of six integral membrane proteins where LTC4S, microsomal glutathione S-transferase (MGST) 2 and FLAP constitute one subgroup, MGST1 and microsomal prostaglandin E_2_ synthase-1 (mPGES-1) constitute a second subgroup and MGST3 constitute a separate branch [9], [10]. mPGES-1 catalyzes the formation of prostaglandin E_2_ [11]. FLAP assists in LTA_4_ biosynthesis and MGST2 probably has a role in LTC_4_ production in cells devoid of LTC4S, such as endothelial cells [12]. MGST1, 2 and 3 have been suggested to be part of a detoxification system catalyzing GSH conjugation reactions to facilitate excretion of xenobiotics [13]. So far all but MGST2 and MGST3 have been structurally characterized.

In previous studies we have characterized the structure and catalysis of human LTC4S (hLTC4S) showing the importance of the amino acid Arg104 that coordinates and stabilizes the glutathionyl thiolate anion prior the conjugation reaction with LTA_4_ to produce LTC_4_ [14]. Furthermore, previous work has shown that the enzyme can bind and stabilize two different GSH conformations. One is “U” shaped, which is suggested to correspond to a substrate mode of conformation prior to catalysis [15]. Recently we identified a second, more relaxed GSH conformation, in S-hexyl GSH, representing a product conformation suggested to exist prior to product release [16].

**Figure 2:**
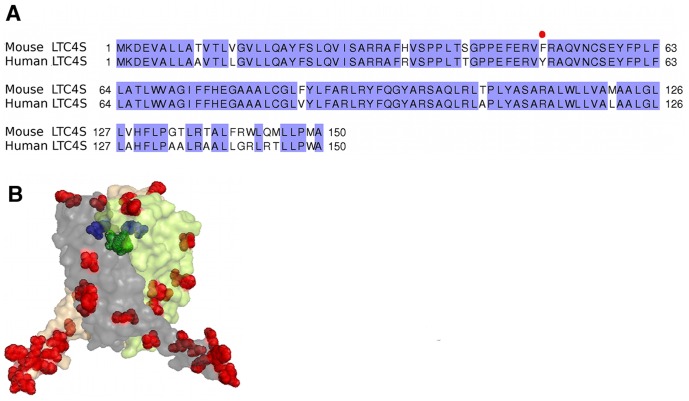
Comparison of human and mouse LTC4S enzymes. A. Amino acid sequence alignment of human and mouse LTC4S generated with the program ClustalW. Species differences are highlighted in white. B. Mapping the amino acid differences (in red) between mouse and human trimeric LTC4S structures. The active site in one monomer is depicted with a bound GSH (green). In blue is the Phe50Tyr exchange positioned close to the active site.

Human LTC4S is 85% identical at the amino acid level to its mouse counterpart (Figure 2), which would suggest that these enzymes have similar structural and biochemical properties [17]. However, small sequence differences can generate large differences in activity and inhibitor binding properties as seen for example with the related mPGES-1 enzyme, where inhibitor efficiency differed substantially between the rat and human orthologs although the enzymes display a sequence identity of 77% [18]. In light of these circumstances and the need to use mouse disease models for development of drugs targeted against LTC4S, we solved the crystal structure of mLTC4S and carried out a kinetic characterization of the enzyme for direct comparison with the structural and catalytic properties of hLTC4S. In addition, we synthesized and tested a nM inhibitor against both the mouse and human isoenzymes.

## Materials and Methods

### Materials

Dodecyl maltoside was obtained from Anatrace. LTA_4_ methyl ester (BIOMOL) in tetrahydrofuran was saponified with 1 M LiOH (6%, v/v) for 48 h at 4°C. All other chemicals were obtained from common commercial sources. S-Hexyl GSH was synthesized as described in [16].

### Cloning and Plasmid Construction

The mouse *LTC4S* cDNA (NM_008521.1, Origene Technologies) was sub-cloned into pPICZA (Invitrogen). Both the cDNA, supplemented with an N-terminal sequence encoding a His_6_ tag, and the vector were PCR amplified and the products were co-transformed into CaCl_2_-competent *E. coli* (TOP10, Invitrogen) using the endogenous recombinase activity of *E. coli* to recombine the fragments. Primers used for recombination were: 5′CGACAACTTGAGAAGATCAAAAT GTCTCACCATCATCACCACCATAAGGACGAAGTGGCTCTTCTGGCT-3′ and 5′-GCA AGACCGGTCTTCTCCCTCAGGCCATCGGCAGGA-3′. The protein coding part of the resulting plasmid, pPICZ-hisLTC4S, was verified by DNA sequencing (SEQLAB, Göttingen, Germany).

### Protein Expression and Purification

The expression vector was transformed into *P. pastoris* KM71H cells using the Pichia EasyComp Transformation kit (Invitrogen). The protein was expressed and purified from *Pichia pastoris* as previously described [15]. The purified protein was either stored frozen at –20°C or directly further polished in a buffer exchange step on a Superdex 200 16/60 (GE Healthcare) equilibrated with 0.03% w/v DDM (w/v), 20 mM Tris pH 8.0, 100 mM NaCl and 0.5 mM TCEP. Fractions containing mLTC4S were concentrated to 3.5 mg ml^−1^ by ultrafiltration and used for setting-up crystallization and activity assays.

### Synthesis of TK04

The synthesis of the inhibitor, (2-Benzoyl-5-{5-[(4-chlorophenyl) (methyl)amino]pyridine-2-carbonyl}benzoic acid), here referred to as “TK04” was prepared with standard procedures according to Nilsson, P. et al. [19].

### Enzyme Kinetics

Enzyme activity towards GSH and LTA_4_ for mLTC4S was determined with aliquots of enzyme (0.1 µg) diluted to 100 µl with 25 mM Tris-HCl (pH 7.8) supplemented with 0.05% Triton X-100. To determine the kinetic parameters for GSH, the concentration of LTA_4_ was kept constant at 36 µM. To determine the kinetic parameters for LTA_4_, the concentration of GSH was kept constant at 5 mM. The incubations were performed on ice essentially as described in Rinaldo-Matthis et al. [14]. Prostaglandin B_2_ (620 pmol) was added as an internal standard before reversed phase-HPLC. The amount of LTC_4_ was quantified by calculating the ratio of the peak area compared with the internal standard prostaglandin B_2_. The *k*_cat_ and *K*_m_ (Table 1) values were determined from the initial velocity of the LTC4S-catalyzed reaction measured as a function of substrate (GSH or LTA_4_) concentrations. The initial velocity data were fitted to the Michaelis-Menten equation using GraphPad Prism 4.

**Table 1:** Data collection, refinement and model building statistics of mLTC4S in complex with sulfate (apo-form), GSH and S-hexyl GSH.

Data set	SO_4_^2−^ (Apo-form)		GSH	S-hexyl GSH	
**Data collection**					
Wavelength (Å)	0.96863		0.93990	0.93928	
Resolution range (Å)	42.39–2.7 (2.85–2.7)		28.2–2.7 (2.85–2.7)	48.82–2.65(2.79–2.65)	
Unit-cell parameters (Å,°)	*a* = *b* = *c* = 169.6, *α* = *β* = *γ* = 90		*a* = *b* = *c* = 169.3, *α* = *β* = *γ* = 90	*a* = *b* = *c* = 169.1 *α* = *β* = *γ* = 90	
Space group	*F*23		*F*23	*F*23	
Observed reflections	46866		143931	158139	
Unique reflections	17347		27094	19576	
Completeness (%)	99.5 (99.0)		100 (100)	100 (100)	
<I*/σ*(I)>	14.0 (0.9)		21.3 (0.6)	24.7 (0.5)	
*R*_merge_ a (%)	4.6 (82)		7.9 (129)	6.0 (157)	
Average multiplicity	3.3		11.1	10.9	
Wilson B-factor (Å^2^)	78.62		68.64	74.7	
**Refinement**					
Reflections used in working set	11160		11164	11766	
Reflections used in test set	558		558	584	
Maximum resolution (Å)	2.7		2.7	2.65	
*R_work_* b/*R_free_* c (%)	23/26		21.3/24	21.7/24.5	
No. of protein atoms	1181		1172	1157	
No. of ligand atoms, ions	49		54	72	
Average B-factor (Å^2^)	83.59		77.76	82.93	
**R.m.s.d. from ideal**					
Bond lengths (Å)	0.010		0.010	0.010	
Bond angles (°)	1.284		1.278	1.367	
**Ramachandran statistcs of φ/ψ angels** d **(%)**					
Preferred regions	91.78	95.17			95.8
Allowed regions	7.56	6.78			8.62
Outliers	1.37	1.38			1.4
**PDB ID**	4NTA	4NTB			4NTF

a *R*_merge_ = ∑_hkl_∑_i_|I_i_(*hkl*) – <I(*hkl*)>|/∑_hkl_∑_i_|I_i_(*hkl*)|, where I_i_(*hkl*) is the intensity of the *i*th measurement of reflection *hkl* and <I(*hkl*)> is the average intensity of this reflection.

b *R* = ∑ ||F_obs_|–|F_calc_||/∑ |F_obs_|.

c *R_free_* [38] was monitored with 5% of the reflection data excluded from refinement.

d as determined by *MolProbity.*

Values for the highest resolution shell are given in parentheses.

### Enzyme Inhibition Assay

For determination of IC_50_ values of inhibitor TK04 against mLTC4S and hLTC4S, a 96-well format assay was used. The assay was carried out on a Polypropylene 96-well plate. A 2 mM solution of LTA_4_ in ACN/borate buffer (1∶1, pH 10) was prepared freshly. The test solution contained 0.1 µg mouse or human LTC4S diluted to 100 µl with 25 mM Tris-HCl (pH 7.8) supplemented with 0.05% Triton X-100 and 5 mM GSH. Two µl of TK04 in DMSO (final concentration 0.1–15 µM) was added and the mixture was incubated for 30 min on ice. To start the reaction, 1 µl of LTA_4_ (final concentration 20 µM) was added and the reaction was quenched after 15 s by the addition of 200 µl methanol. Prostaglandin B_2_ (496 pmol) was added as an internal standard before reversed phase-HPLC, which was performed on the same HPLC system as described above. GraphPad Prism 4 was used to calculate the kinetic parameters IC_50_ and *K*_i_. IC_50_ was determined using the equation Y = 100/(1+10^(X−LogIC50)^) and *K_i_* was determined using the Michaelis−Menten equation edited for competitive inhibition. All measurements were done in triplicates.

### Crystallization

The crystals for the mLTC4S were grown and cryo-cooled essentially as described in Niegowski et al. [16]. To obtain the apo (SO_4_^2−^) and GSH crystal complexes, 1 µl of protein solution supplemented with or without 1 mM GSH was mixed with 1 µl of reservoir solution containing 1.8–2.2 M NH_4_SO_4_, 0.2 M NaCl and 0.1 M Na cacodylate pH 6.1–6.8, and cryo-cooled. To obtain the S-hexyl GSH complex, crystals were obtained as described above, without GSH, and soaks were conducted in the reservoir solution with the addition of 1 mM S-hexyl GSH in time intervals ranging from 30 seconds to 24 hours.

### Data Processing, Structure Solution and Refinement

Data were collected at the ESRF beamline ID29 (mLTC4S in complex with SO_4_^2−^ and S-hexyl GSH) and at the Diamond beamline I24 (mLTC4S in complex with GSH). The data were processed using XDS and scaled with SCALA [20], [21]. Data cutoff was chosen with the new assessment criteria based on the correlation coefficient (CC) described by Karplus, A.P. et al. [22]. The structure was solved using molecular replacement with PHASER using a modified PDB ID 2UUI with waters and lipids removed [23]. Refinement and simulated annealing was carried out with REFMAC and the PHENIX suite [24], [25]. In order to avoid model bias, 25 cycles of simulated annealing were carried out prior to model building and ligand introduction with a starting temperature of 5000 K. Model building was done using Coot [26]. All structure figures were produced using PYMOL [27]. X-ray statistics are presented in Table 2.

**Table 2:** Steady state kinetic parameters of mLTC4S and hLTC4S against GSH and LTA_4_.

	mLTC4S			hLTC4S		
Substrate	*k*_cat_ (s^−1^)	*K*_M_ (µM)	*k*_cat_/*K*_M_(s^−1^ M^−1^)	*k*_cat_ (s^−1^)	*K*_M_ (µM)	*k*_cat_/*K*_M_(s^−1^ M^−1^)
LTA_4_	81±7.0	36±8	(2.3±0.26)×10^6^	15.5±0.8	23±3	(0.6±0.057)×10^6^
GSH	84±5.7	1200±240	(6.8±0.8)×10^4^	12±0.7	300±60	(4.0±1.1)×10^4^ **

The enzyme activity was measured in 25mM Tris (pH 7.8), 0.1M NaCl, 0.05% DDM in the presence of either 30 µM LTA_4_ and/or 5 mM GSH with 0.1 µg of enzyme.

**[29].

## Results and Discussion

Since LTC4S is an integral membrane protein it was a major breakthrough when its crystal structure was recently solved at high resolution and details of its catalytic mechanism uncovered [15], [16], [28], [29]. In the present report we continue this work and describe the structure of mLTC4S. The enzymatic properties of mLTC4 were determined and we compared in parallel the mouse and the human enzymes’ ability to bind a potent LTC4S inhibitor.

### Cloning and Expression of mLTC4S

The mLTC4S cDNA with an N-terminal hexa-histidine tag was cloned into the expression vector pPICZA, subsequently used to transform Pichia cells for over-expression. From a 5 l culture, around 3–5 mg of protein was obtained. The recombinant mLTC4S was purified using similar methods as described for the human enzyme [14]. The enzyme was 90% pure as evaluated with SDS page. The protein was stable in the presence of 1 mM GSH and 0.05% DDM and the same enzymatic activity was maintained over a 24 h period when stored at 4°C.

### Structure of Mouse LTC4S

Purified recombinant mLTC4S was crystallized and structurally characterized. The three structures of mLTC4S presented here were solved in complex with a sulfate ion, GSH and S-hexyl GSH, to a resolution of 2.65 Å, 2.7 Å and 2.65 Å respectively (Table 1). The overall structure of the biologically active homotrimer of mLTC4S was similar to previously solved structures of the human enzyme [30], [31], [32]. The structures were solved with one monomer in the asymmetric unit, where each monomer has four transmembrane helices (helices I, II, III and IV) and one helix (V) that extrudes into the solvent. The active site is situated in the interface between neighboring monomers, facing the membrane side essentially as described for hLTC4S [16].

**Figure 3:**
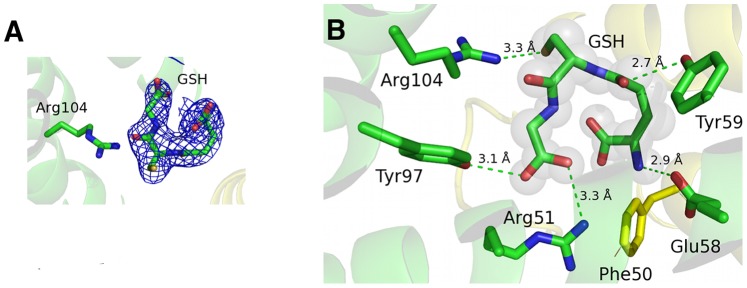
Binding of GSH at the active site of mLTC4S. A. Electron density 2fo-fc map contoured at 1.0 σ around GSH with Arg104 coordinating the sulfur in GSH. B. GSH bound at the active site, coordinated by several amino acids where the Arg51 - Tyr50 (indicated with a line) interaction in the human enzyme, is lost in the mLTC4S, which has a Phe in position 50.

**Figure 4:**
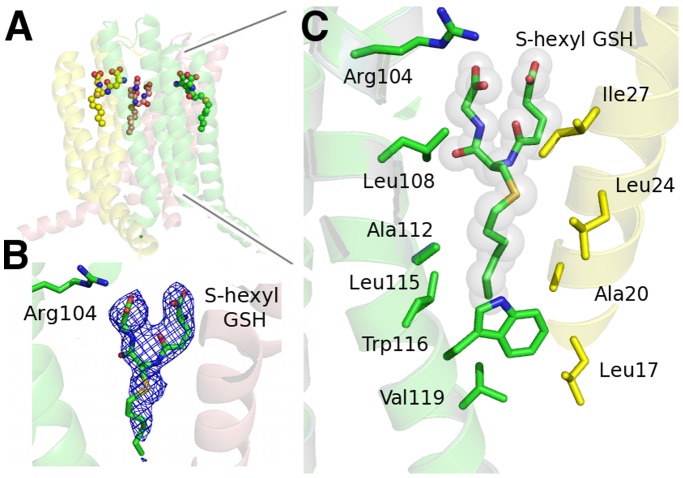
Binding of S-hexyl GSH to the active site of mLTC4S. A. The trimeric form of mLTC4S with three bound S-hexyl GSH. B. Electron density 2fo-fc map, contoured at 1.0 σ around S-hexyl GSH. C. The hydrophobic cavity with S-hexyl GSH bound (yellow stick carbons) in the hydrophobic cleft. Amino acids facing the cavity are from monomers A (yellow) and B (green).

The ligands GSH and S-hexyl GSH were identified as strong positive residual density in the Fo–Fc maps after simulated annealing and initial refinement but before ligand introduction. The 2Fo–Fc maps with ligands after refinement are shown in Fig. 3A and 4B.

In the structure of the mLTC4S in complex with GSH, the tripeptide has a “U” shaped conformation coordinated in a similar way as described in [15], [16] with Arg104 interacting (distance of 3.3 Å) with the glutathione thiolate (Figure 3). Arg104 has previously been identified as a catalytic residue, which stabilizes and probably also induces formation of the thiolate anion [14].

The product mimic, S-hexyl GSH (Figure 1B) was soaked with mLTC4S crystals generating a structure complex with S-hexyl GSH bound (Figure 4). The S-hexyl GSH binds with its GSH moiety at the GSH-binding site and with its alkyl chain in a cavity lined by several hydrophobic amino acids such as Leu108, Ala112, Leu115, Trp116, Val119 and from the neighboring subunit Leu17, Ala20, Leu24 and Ile27. The alkyl chain overlaps partly with the DDM molecule bound in the hLTC4S (2UUH) structure. The S-hexyl GSH binds in an “extended” or relaxed conformation [16] as compared to GSH in the GSH structure complex and the shift in position of the sulfur of S-hexyl GSH increases the distance to Arg104 from 3.3 Å in the GSH structure to about 9 Å, a distance not compatible with any important interaction, also reflected in a substantially lower binding affinity of S-hexyl GSH with a *K*_i_ of about 2 mM [16] as compared with GSH (*K*_d_ = 14 µM) [29]. The conformation of the S-hexyl GSH is suggested to represent a state of product release.

**Figure 5:**
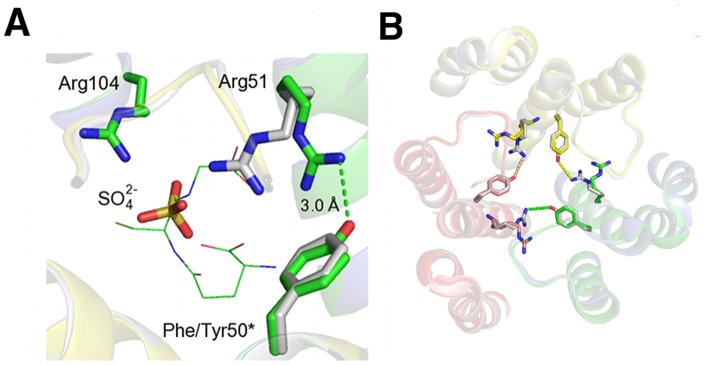
Positional shift of Arg51 and loss of salt bridge at the active site of mLTC4S. A. Close up of the mLTC4S complex with SO_4_^2−^, showing a shift in the position of Arg51 due to Phe50Tyr exchange. Human LTC4S is colored in green and mLTC4S is colored in gray. GSH is shown as green “lines”. *indicates that it is positioned on the neighboring subunit. B. Trimer of mLTC4S showing the amino acid exchange at position 50 where Phe in mLTC4S fails to make a salt bridge with Arg51. In hLTC4S, the Tyr50-Arg51 couple will likely contribute to trimer stability.

Almost all residues that interact with GSH are similar between the two enzymes. However one interesting difference involves Arg51, a key active site residue that makes a salt bridge to one carboxyl end of GSH. In hLTC4S, Arg51 is held in position by the second sphere residue Tyr50 that makes a salt bridge to Arg51, an interaction that might be important for trimerization of the protein (Figure 5). In mLTC4S, Tyr50 has been exchanged for a Phe, which prevents formation of a salt bridge with Arg51. In the structure complex of mLTC4S with SO_4_^2−^, Arg51 has therefore shifted position as compared to Arg51 in hLTC4S. The slightly higher *K*_m_ as seen for the mLTC4S (*K*_m_^GSH^ = 1.2±0.24 mM) as compared to the human enzyme (*K*_m_^GSH^ = 0.3±0.06 mM) might be explained by a less stable active site conformation in mLTC4S enzyme due to Tyr50Phe exchange. In this context, it is interesting to note that 9 of the 18 amino acids that differ between the two enzymes, are situated at the carboxy-terminus, and studies by Svartz et al. [33] have shown that the carboxy terminal α-helix V has a special role in oligomerization of LTC4S (Figure 2B).

### Catalytic Efficiency of mLTC4S

The purified recombinant mLTC4S was active, readily catalyzing conjugation of GSH with LTA_4_, as assessed by reverse-phase HPLC. Varying LTA_4_ (1–100 µM) and measuring steady state kinetic parameters of LTC_4_ production, we determined a catalytic efficiency, *k*_cat_/*K*_m_, of 2.3±0.26×10^6^ s^−1^ M^−1^ for the mouse enzyme and for the human enzyme, the *k*_cat_/*K*_m_ was 0.64±0.057×10^6 ^s^−1^ M^−1^. The binding affinity for LTA_4_, measured as *K*_m_^LTA4^, were in the same range, 23±3 µM and 36±8 µM, respectively, for the human and mouse enzyme. Varying GSH (0–8 mM) and keeping the concentration of LTA_4_ constant at 30 µM, we got a catalytic efficiency of 6.8±0.8×10^4^ s^−1 ^M^−1^ for the mouse enzyme, which is similar to the human enzyme 4.0±1.1×10^4 ^s^−1 ^M^−1^ obtained in earlier studies. The *K*_m_^GSH^ was 1.2±0.24 mM for mLTC4S, and 0.3±0.06 mM for hLTC4S (Table 2). These results suggest a similar catalytic mechanism of both enzymes however this needs to be further confirmed.

### Inhibition of Enzyme Activity with LTA_4_ as Substrate

**Figure 6:**
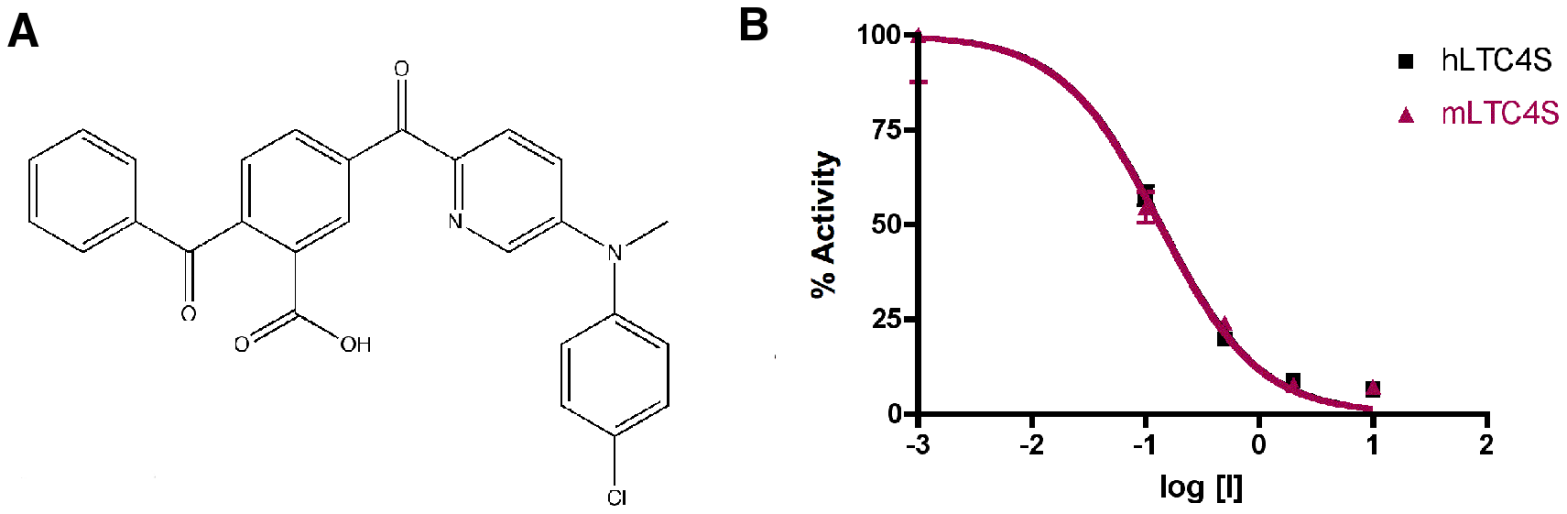
TK04 is a nanomolar competitive inhibitor of LTC4S. A. Chemical structure of TK04, the inhibitor used in this study. Dose-response curves for inhibition of mouse and human LTC4S by TK04. 100% activity corresponds to the enzyme activity without inhibitor, which was 44.0 µmol min^−1^ mg^−1^ for the mouse enzyme (red line) and 69.7 µmol min^−1^ mg^−1^ for the human enzyme (black line). The concentrations of substrates GSH and LTA_4_ used in the assay were 5 mM and 20 µM, respectively. The IC_50_ for mLTC4S was 135±30 nM and for the hLTC4S it was 134±16 nM.

Only very few inhibitors of LTC4S have been reported thus far [34]. In this study we present the inhibitor, TK04 (Figure 6A) that efficiently inhibits both mouse and human LTC4S.

To establish the affinity of TK04 towards mLTC4S and hLTC4S, the inhibitor (0.05–10 µM) was incubated with the enzymes (0.1 µg each) with varying concentrations of LTA_4_ (10, 20 and 40 µM) at a fixed concentration of GSH (5 mM). Formation of LTC_4_ was measured by reverse-phase HPLC. Under these conditions, TK04 potently inhibits both the mouse and the human LTC4S with an IC_50_ of 135±30 nM and 134±16 nM, respectively, with 20 µM LTA_4_ (Figure 6B).

The IC_50_ value of the inhibitor increased with increasing LTA_4_ concentrations, suggesting that the inhibitor and LTA_4_ compete for the same site on the enzyme. To further determine the mode of inhibition, both human and mouse enzyme were treated with TK04 at concentrations between 0.1–10 µM followed by incubation with varied LTA_4_ concentrations (5, 20, 50 and 80 µM). The experimental data was analyzed with nonlinear regression along with the competitive inhibition model using GraphPad Prism 4. The calculated *K*_i_ values of TK04 for the human and the mouse enzyme were 41±6.6 nM and 37±6.4 nM respectively.

The observed competitive mode of binding of TK04 towards LTA_4_ is indicative of the inhibitor having the capacity to bind to the hydrophobic LTA_4_ binding site. Although TK04 was equipotent against the mouse and human enzymes, it is important to keep in mind that this may not be the case for other inhibitors with different chemistry, the binding of which may be influenced by interactions with Arg51. To fully understand the nature of the interactions of inhibitors with LTC4S, crystal structure complexes will be required. Investigating the structure of the FLAP-inhibitor complex (2Q7M.pdb), we can see that the FLAP inhibitor MK591 binds in a similar place as the S-hexyl GSH in the mouse and human LTC4S, *i.e*. in the hydrophobic cleft formed between two monomers, which might indicate a cross reaction of TK04 with FLAP [35]. However, FLAP lacks enzyme activity and there are considerable structural differences between FLAP and LTC4S at this hydrophobic binding site. Hence, the effect of TK04 on FLAP needs to be tested experimentally.

In conclusion, we have solved the crystal structure of mouse LTC4S in complex with its substrate GSH and a product analog S-hexyl GSH. The substrate GSH is coordinated in a “U” shape whereas the S-hexyl GSH is oriented in a so-called “extended” conformation compatible with a product mimic. Furthermore we have synthesized and experimentally evaluated the affinity and binding mode of a competitive inhibitor, which binds in the nanomolar range, both to the human and the mouse enzyme.

Since human LTC4S is considered a promising target for therapy in asthma or cardiovascular disease [36], [37] it is essential that there is a suitable *in vivo* animal model for testing. We have demonstrated in this study that mLTC4S behaves similarly to the human enzyme with regards to structure, activity and binding of one inhibitor *in vitro*, suggesting that it might be a suitable tool for drug development. However, structural differences near the active site and along the C-terminal α-helix V, suggest that the mouse and human LTC4S may exhibit diverging profiles with other inhibitors and may function differently *in vivo*.
