# Correction: Structure and Inhibition of Mouse Leukotriene C4 Synthase

**DOI:** 10.1371/journal.pone.0099747

**Published:** 2014-06-04

**Authors:** The PLOS ONE Staff

The Data Availability statement does not appear on the paper. The correct Data Availability statement is: The authors confirm that all data underlying the findings are freely available without restriction. Data are included in the paper and structural data can be found in the Protein Data Bank under PDB IDs: 4NTA,4NTB and 4NTF.

The publisher apologizes for this error.
